# Lysosomal peptidases—intriguing roles in cancer progression and neurodegeneration

**DOI:** 10.1002/2211-5463.13372

**Published:** 2022-02-03

**Authors:** Janko Kos, Ana Mitrović, Milica Perišić Nanut, Anja Pišlar

**Affiliations:** ^1^ 61790 Faculty of Pharmacy University of Ljubljana Slovenia; ^2^ Department of Biotechnology Jožef Stefan Institute Ljubljana Slovenia

**Keywords:** cancer, cathepsins, lysosomes, neurodegeneration, peptidases

## Abstract

Lysosomal peptidases are hydrolytic enzymes capable of digesting waste proteins that are targeted to lysosomes via endocytosis and autophagy. Besides intracellular protein catabolism, they play more specific roles in several other cellular processes and pathologies, either within lysosomes, upon secretion into the cell cytoplasm or extracellular space, or bound to the plasma membrane. In cancer, lysosomal peptidases are generally associated with disease progression, as they participate in crucial processes leading to changes in cell morphology, signaling, migration, and invasion, and finally metastasis. However, they can also enhance the mechanisms resulting in cancer regression, such as apoptosis of tumor cells or antitumor immune responses. Lysosomal peptidases have also been identified as hallmarks of aging and neurodegeneration, playing roles in oxidative stress, mitochondrial dysfunction, abnormal intercellular communication, dysregulated trafficking, and the deposition of protein aggregates in neuronal cells. Furthermore, deficiencies in lysosomal peptidases may result in other pathological states, such as lysosomal storage disease. The aim of this review was to highlight the role of lysosomal peptidases in particular pathological processes of cancer and neurodegeneration and to address the potential of lysosomal peptidases in diagnosing and treating patients.

## Graphical abstract

Lysosomal peptidases participate in various processes, which may regulate either cancer progression or neurodegeneration.

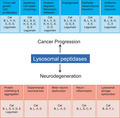

**Abbreviations**
Cat: cathepsin
CDK2‐AP1: cyclin‐dependent kinase 2‐associated protein 1
CDP/Cux: CCAAT‐displacement protein/cut homeobox
ECM: extracellular matrix
EGF: epithelial growth factor
EMT: epithelial–mesenchymal transition
ERK: extracellular signal‐regulated kinase
FAK: focal adhesion kinase
IGF‐I: insulin‐like growth factor I
JNK: c‐Jun N‐terminal kinase
LRP1: low‐density lipoprotein receptor‐related protein 1
MAPK: mitogen‐activated protein kinase
MCP‐1: monocyte chemotactic protein 1
MDSCs: myeloid‐derived suppressor cells
MMPs: matrix metallopeptidases
PAR4: proteinase‐activated receptor 4
PI3K: phosphatidylinositol‐bisphosphate 3‐kinase
RIPK1: receptor‐interacting Ser/Thr protein kinase 1
RPLP0: ribosomal protein P0
TGF‐β: transforming growth factor‐β
TIMPs: tissue inhibitors of metalloproteases
TLR: Toll‐like receptor
TNF‐α: tumor necrosis factor α
TRAIL: tumor necrosis factor‐related apoptosis‐inducing ligand

Lysosomes are membrane‐bound organelles that are found in most cells. They were discovered and named by Christian de Duve (reviewed in [1]) and later recognized as the main waste disposal system of the cell, digesting both intracellular and extracellular materials [2]. Lysosomes have a diameter of 0.1–1.2 μm and a pH of 4.5–5.0 [3]. The two main pathways of waste entry into lysosomes are endocytosis and autophagy, which internalize extracellular and intracellular material, respectively.

During endocytosis, a part of the cell's plasma membrane forms vesicles that embed extracellular material. These vesicles arise at the plasma membrane through a variety of mechanisms [4, 5]. Clathrin‐dependent endocytosis accounts for the formation of most endocytic vesicles. It involves binding between the clathrin and cytoplasmic domains of plasma membrane proteins, formation of clathrin‐coated pits, and budding of clathrin‐coated vesicles. Clathrin‐coated vesicles are internalized and then fuse with specific acceptor compartments [6]. Conversely, clathrin‐independent endocytosis comprises several internalization mechanisms, of which the most recognized are caveola‐dependent [7] and actin‐driven [8]. Furthermore, micropinocytosis is another mechanism of endocytosis that occurs in highly ruffled regions, forming actin extensions around extracellular fluid [9]. All endocytosed materials first reach early endosomes, in which they are sorted either for recycling or degradation. Transport toward lysosomes involves globular late endosome intermediates, also called multivesicular bodies [10].

Autophagy represents the uptake of intracellular components into endo/lysosomal compartments. Autophagy is divided into different types, including macroautophagy [11], microautophagy [12], chaperone‐mediated autophagy [13], and various types of selective autophagy leading to the destruction of particular organelles.

Normal lysosomal function is enabled through the actions of two classes of proteins: lysosomal membrane proteins and lysosomal enzymes, predominantly soluble lysosomal hydrolases (also referred to as acid hydrolases). Approximately 50 lysosomal membrane proteins have been described so far. Soluble lysosomal hydrolases constitute the main degradative lysosomal component, and to date, more than 50 hydrolases and their accessory proteins have been detected. Lysosomal hydrolases comprise a variety of peptidases, nucleases, glycosidases, sulfatases, and lipases [14]. The aim of this review was to highlight the role of lysosomal peptidases, in particular lysosomal cathepsins, in the pathological processes of cancer and neurodegeneration.

## Lysosomal peptidases

Peptidases represent a large family of lysosomal hydrolases, which are present in all living organisms and catalyze the hydrolysis of peptide bonds in different biological processes. Almost 600 human peptidases are listed in the MEROPS database and can be classified into seven main groups according to their structure and catalytic type: serine, cysteine, threonine, and aspartyl, glutamyl, asparaginyl, and metallopeptidases [15]. Lysosomal peptidases are involved in the complete breakdown of proteins targeted to lysosomes; however, they may also have other regulatory functions either within or outside of lysosomes.

In mammalian cells, the most predominant lysosomal peptidases can be grouped into four major families: aspartic cathepsins (D and E), serine cathepsins (A and G), the asparagine endopeptidase legumain (a cysteine peptidase with a fold more related to caspases than family C1 peptidases [16]), and cysteine cathepsins (B, C, F, H, K, L, O, S, V, W, and X/Z), annotated as clan CA, family C1a [17].

The serine peptidase cathepsin (Cat) A is involved in regulating the activity of lysosomes, stability of lysosomal glycosidases (e.g., beta d‐galactosidase and *N*‐acetyl‐alpha‐neuraminidase), and transport of neuraminidase to mature lysosomes [18]. In addition, this serine peptidase triggers the degradation of LAMP‐2A, a receptor involved in chaperon‐mediated autophagy [18].

Another serine peptidase, Cat G (CatG), is known as the main granule‐associated proteolytic enzyme of neutrophils [19]. However, it is also found in the endo/lysosomal compartments of a variety of antigen‐presenting cells, in which it plays a critical role in antigen and autoantigen processing [20]. For example, CatG is involved in proteolytic cleavage, subsequent activation of chemokines, cytokines, and cell surface receptors, antigen processing, and clearance of internalized pathogens [21, 22, 23]. As such, CatG is involved in the defense against invading pathogens. However, its dysregulated proteolytic activity contributes to pathological conditions such as chronic pulmonary diseases, human immunodeficiency virus infection, tumor progression, and metastasis (extensively reviewed in [20, 24]).

Cathepsin E (CatE) is an aspartic peptidase and a member of the A1 peptidase family, along with pepsin A and Cat D (CatD). CatE is expressed intracellularly in gastrointestinal cells, immune cells, and several cell types within lymphoid tissues [25, 26]. In antigen‐presenting cells, CatE is located in endo/lysosomal compartments and is most likely involved in the breakdown of antigenic proteins [27]. However, in erythrocytes and gastric cells, CatE is expressed in the plasma membrane [27, 28, 29], and its function is not fully understood. Furthermore, a recent study has shown it may be an early biomarker for certain types of cancer [25].

The most abundant aspartic lysosomal peptidase is CatD [30]. Through its endopeptidase activity, CatD modulates the activity of diverse polypeptides, growth factors, and enzymes and the degradation of misfolded, long‐lived, and denatured proteins. Accordingly, CatD is considered an essential regulator of cell signaling and cellular homeostasis [31]. Dysregulated CatD activity plays an important role in diseases such as acute kidney injury, coronary events, neurodegenerative diseases, and cancer (reviewed in [31, 32, 33, 34]).

Legumain (also named asparagine endopeptidase; clan CD, family C13) is named after its strict specificity for cleavage of asparagine residues and was classified as a member of the C13 family of cysteine peptidases (EC 3.4.22.34) [35]. Legumain is predominantly located in late endo/lysosomes of antigen‐presenting cells, such as B cells and dendritic cells. In B cells, legumain participates in processing endogenous and foreign proteins for presentation of the major histocompatibility complex class II molecules on the surface of T cells [36], whereas in dendritic cells, legumain plays an indispensable role in activating Toll‐like receptor (TLR) 9, which is critical for full cytokine production [37]. Legumain was reported to regulate the stability of FOXP3, a transcription factor that controls the immunosuppressive program in CD4^+^ T cells [38]. It also plays a role in osteoclast formation and bone resorption (by regulating the differentiation fate of human bone marrow stromal cells) and in extracellular matrix (ECM) remodeling in kidneys, lung, liver, and pancreas (reviewed in [16]). Finally, dysregulated legumain activity is associated with cancer and neurodegenerative diseases, including Alzheimer's disease (AD), stroke, ischemia, amyotrophic lateral sclerosis (ALS), and multiple sclerosis [39, 40].

The fourth family consists of papain‐like cysteine peptidases, which are the main focus of this review. They represent the largest family of cathepsins, with 11 cysteine cathepsins encoded in the human genome (B, C/DPP1, F, H, K, L, O, S, W, V, and X/Z). Some cysteine cathepsins, such as cathepsins B, H, and L, are ubiquitously expressed in human tissues and represent enzymes with broad substrate specificities. However, certain cysteine cathepsins (e.g., S, X, V, K, and W) are strictly expressed in specific cell types (reviewed in [41]). Most of them exhibit endopeptidase activity (by cleaving internal peptide bonds), whereas only a few exhibit exopeptidase activity and possess additional structural elements that restrict access to the active site and form electrostatic bonds with the C or N termini of substrates [42, 43]. Due to these structural variances, cathepsins B (CatB) and X (CatX; also known as Cat Z, P, IV/B2/Y, and lysosomal carboxypeptidase B) can act as dipeptidyl carboxypeptidases and carboxymonopeptidases, respectively [44, 45], whereas cathepsins C (CatC; also known as dipeptidyl peptidase I) and H (CatH) cleave their substrates as aminopeptidases [15, 46]. Only CatB and CatH exhibit both endopeptidase and exopeptidase activities, depending on their localization, that is, the pH of the environment [47, 48].

In specialized immune cells, such as cytotoxic T lymphocytes (CTLs) and natural killer (NK) cells, several other peptidases can be found in the endo/lysosomal pathway. These cells contain secretory lysosomes, that is, cytotoxic granules, which are exocytosed during specific interaction with target cells. Cytotoxic granules contain serine peptidase granzymes and perforin, which, together with cysteine cathepsins, trigger apoptosis in target cells [49].

The activity of cathepsins is controlled by different mechanisms, which include peptidase expression (regulated at the transcriptional and translational levels), cofactors, lysosomal trafficking, the specificity of the active site cleft, and pH. Furthermore, cathepsins are synthesized and delivered to early lysosomes as inactive precursors, which are further activated either by lower pH, proteolytic processing by other endo/lysosomal hydrolases, or interaction with glycosaminoglycans [50, 51, 52, 53, 54, 55, 56]. Cathepsin activity was examined in various kinetic studies using specific substrates and visualized by fluorescently labeled activity‐based probes both *in vitro* and *in vivo* [57, 58, 59, 60]. Ultimately, endogenous protein inhibitors regulate the activity of mature cathepsins that escape endo/lysosomal vesicles and are present in the cytoplasm or extracellular space or bound to the plasma membrane. Several groups of endogenous inhibitors of cysteine, serine, and metallopeptidases have been shown to impair secreted or misdirected lysosomal cathepsins, including cystatins, serpins, and tissue inhibitors of metallopeptidases [61, 62]. Nevertheless, certain exceptions, such as cystatins M and F, can enter the endo/lysosomal pathway and regulate intralysosomal peptidase activity [62].

Lysosomal cathepsins were long believed to participate in only intracellular protein turnover; however, later, they were found to also play roles in a number of other physiological and pathological processes [63]. For example, extralysosomal cathepsins have been associated with prohormone activation, apoptosis, cell migration, cancer, and neurodegeneration [64, 65].

## Lysosomal peptidases in cancer progression

### Lysosomal peptidases in cancer cell signaling

The development and progression of cancer is a complex multistep process of genetic and epigenetic alterations that drive the transformation of normal cells to their malignant forms [66, 67]. The transformation is often followed by dysregulation of lysosomal proteolytic enzymes involved in signaling pathways in transformed or malignant cells [50, 68, 69]. Lysosomal peptidases interfere with cytokine/chemokine signaling and modify growth factors and receptors crucial for tumor cell growth and proliferation (Table 1; reviewed in [68, 70]). CatB participates in the signaling of activators of proliferating cells, such as insulin‐like growth factor I (IGF‐I) and transforming growth factor‐β (TGF‐β). IGF‐I is important for regulating and maintaining the invasive and metastatic properties of the malignant phenotype [71], whereas TGF‐β exhibits both tumor‐suppressive and tumor‐promotive properties [72] and is a key regulator of the epithelial–mesenchymal transition (EMT). CatB regulates the production and signaling of TGF‐β by direct activation [73, 74] or by ECM proteolysis and subsequent TGF‐β release [75]. The downregulation of CatB (both by silencing and inhibition) reduces TGF‐β signaling and invasion [73, 76]. CatB is also responsible for the degradation of epithelial growth factor (EGF) and its internalized receptor complex, as observed in thyroid cancer, glioma cells, and liver [77, 78].

**Table 1:** Significance of lysosomal peptidases in cancer.

Type of Cat	Function	Role in cancer	References
CatB	IGF‐I, EGF, TGF‐β signaling	Tumor cell growth, cell proliferation, invasion, EMT, angiogenesis	[71, 73, 74, 77, 78, 142]
	Regulation of MAPK/ERK and PI3K/Akt signaling, TLR3	Tumor progression	[79, 80, 81, 141]
	Cleavage of cell cycle inhibitor p27^Kip1^	Tumor cell proliferation	[82]
	Cleavage of Bid, Bcl‐2 family proteins, lipid signaling enzyme sphingosine kinase 1, RIPK1	Apoptosis, cell death	[124, 125, 126, 128, 129, 130]
	Degradation of ECM proteins	Invasion, metastasis, angiogenesis	Reviewed in [56]
	Induction of EMT, regulation of EMT markers	EMT, invasion	[142, 143, 145]
	Degradation of angiogenesis inhibitors and release of growth factors	Angiogenesis	[75, 182, 244]
	MDSC function and activity	Tumor progression, suppression of antitumor immunity	[235]
CatL	EGF, TGF‐β processing and signaling	Tumor cell growth, EMT, invasion	[83, 147]
	Activation of MAPK/ERK and PI3K/Akt signaling	Angiogenesis, EMT, invasion	[84, 85, 148, 149]
	CDP/Cux and 53DP1 processing	Tumor cell proliferation, EMT, angiogenesis	[84, 88, 89, 91, 92, 93]
	Cleavage of Bid, Bcl‐2 family proteins, complement and CDK2‐AP1	Apoptosis, cell proliferation	[86, 125, 126, 131, 215]
	Degradation of ECM proteins	Invasion, metastasis, angiogenesis	Reviewed in [90, 146]
	Induction of EMT, regulation of EMT markers	EMT, invasion	[145, 147, 148, 149, 151]
	Release of growth factors, endothelial cell infiltration	Angiogenesis	[183] Reviewed in [90]
	C‐terminal processing of perforin	Granzyme‐mediated apoptosis	[197]
	Th17 subset and MDSC differentiation	Suppression of antitumor immunity	[219, 233]
CatV	Suppression of GATA3 expression	Hyperproliferation	[94, 95]
	Regulation of epithelial and mesenchymal markers	EMT, invasion	[152]
	Elastin degradation	Invasion, metastasis	[153]
CatS	Regulation of PI3K/Akt/mTOR, JNK, ERK/MAPK, TGF‐β signaling	Autophagy, EMT, invasion	[97, 99, 156]
	Cleavage of Bid, Bcl‐2 family proteins, RIPK1, CD74	Apoptosis	[125, 126, 127, 129, 243]
	Degradation of ECM proteins	Invasion, metastasis, angiogenesis	[154]
	Regulation of EMT markers	EMT, invasion	[145, 155]
	Generation of anti‐ and proangiogenic peptides	Angiogenesis	[184] [154]
	Th17 subset differentiation	Suppression of antitumor immunity	[221]
CatK	Regulation of TLR, Notch signaling, cytokines	Tumor progression, cellular crosstalk, inflammation	[100, 101, 238, 240, 241] Reviewed in [158]
	Cleavage of Bid, Bcl‐2 family proteins	Apoptosis	[125, 126]
	Degradation of ECM proteins, resorption of bone matrix	Invasion, metastasis, angiogenesis	Reviewed in [146, 158] [104]
CatH	Cleavage of Bid, Bcl‐2 family proteins	Apoptosis	[125, 126]
	Talin processing, functional development of tumor vasculature	Migration, adhesion, angiogenesis	[157, 185]
	N‐terminal processing and activation of granzymes	Granzyme‐mediated apoptosis	[200]
CatC	Interaction with TNF‐α/p38 MAPK, JNK signaling	Cell proliferation, metastasis, apoptosis, autophagy	[105, 133]
	N‐terminal processing and activation of granzymes	Granzyme‐mediated apoptosis	[199]
CatX	Regulation of MAPK/ERK, PI3K/Akt, FAK/Src, IGF‐I signaling, RPLP0	Tumor progression, migration, apoptosis	[68, 106, 108, 134]
	Integrin receptor signaling, cleavage of profilin‐1	Adhesion, migration	Reviewed in [107], [163, 164]
	Compensation of CatB proteolytic activity	Invasion, metastasis	[163, 164]
	Bypassing senescence	Tumor progression	[165]
	Induction of EMT, regulation of EMT markers	EMT, invasion, migration	[142, 166]
	MDSC differentiation	Suppression of antitumor immunity tumor progression	[233]
Legumain	Regulation of PI3K/Akt, MYC, p53 signaling	Cell proliferation, apoptosis	[109, 110, 373]
	Interaction with integrin receptors	Cell proliferation, metastasis, migration, EMT	[111, 112, 113]
	Producing the mature forms of MMPs and cathepsins	Invasion, metastasis, angiogenesis	[168, 169]
	Induction of EMT, regulation of EMT markers	EMT, invasion	[170]
	Processing of CatL and Th17 subset differentiation	Suppression of antitumor immunity	[217]
CatD	Interaction with IGF‐II receptor, LRP1‐regulated intermembrane proteolysis	Tumor cell growth, cell proliferation	[115, 118]
	Regulation of ERK and PI3K/Akt in signaling	Tumor cell proliferation, migration, angiogenesis	[116, 117]
	Activation of Bix, caspase‐8	Apoptosis	[114, 135]
	Affecting the fusion of autophagosomes and lysosomes	Apoptosis, autophagy	[137]
	Degradation of ECM proteins	Invasion, migration, metastasis, angiogenesis	[114]
	Cleavage of peptidases and their endogenous inhibitors	Invasion, migration, metastasis	[75, 171, 172, 173]
	Releasing growth factors from the ECM proteins and degradation of antiangiogenic factors	Angiogenesis	[114, 187, 188]
CatG	IGF‐I, TGF‐β signaling	Tumor cell growth, bone resorption, angiogenesis, cell aggregation	[119, 120, 121]
	Downregulation of survivin expression	Apoptosis	[138]
	Regulation of VEGF, MCP‐1, PAR4, cell surface proteins	Invasion, migration, angiogenesis	[178, 180, 190]
CatE	Induction of EMT, regulation of EMT markers	EMT	[175]
	Release of soluble TRAIL	Growth arrest, apoptosis	[176]
	Upregulation of antiangiogenic mediators	Angiogenesis	[189]

Furthermore, CatB mediates tumor progression by regulating kinases involved in Ras/mitogen‐activated protein kinase (MAPK)/extracellular signal‐regulated kinase (ERK) signaling. Loss of CatB was shown to downregulate the MAPK/ERK pathway in pancreatic cancer [79]. Similarly, in glioma cells, CatB regulates cell migration through c‐Jun N‐terminal kinase (JNK), another member of the MAPK family [80]. CatB also regulates phosphatidylinositol‐bisphosphate 3‐kinase (PI3K)/Akt signaling, another pathway that is crucial for tumor progression. Reduced activation of PI3K/Akt signaling was demonstrated in gliomas after CatB downregulation [81]. CatB also promotes tumor cell proliferation by cleaving cell cycle inhibitor p27^Kip1^; higher p27^Kip1^ levels, followed by increased cyclin B1 levels, were observed in CatB‐deficient colorectal tumors [82].

Another lysosomal cysteine Cat involved in cancer cell signaling is Cat L (CatL). During tumor growth, it is responsible for cleaving EGF receptor and consequently activating downstream signaling pathways [68, 83]. Interestingly, CatL‐deficient mouse keratinocytes exhibited increased activation of MAPK/ERK and PI3K/Akt signaling pathways and elevated levels of active Ras [84]. Ras is one of the central molecules in several cancer‐promoting signaling pathways, such as MAPK and Akt [84]. In human omental microvascular endothelial cells, CatL activated the ERK pathway and induced angiogenesis [85]. During cell cycle progression, CatL interacts with cell cycle regulator cyclin‐dependent kinase 2‐associated protein 1 [86], a growth suppressor that negatively regulates cyclin‐dependent kinase 2 [87]. In cancer cells, CatL is also localized in the nucleus. Nuclear CatL processes the CCAAT‐displacement protein/cut homeobox (CDP/Cux) transcription factor to enhance DNA binding [88, 89]. CDP/Cux promotes tumor cell proliferation by accelerating cell entry into the S phase of the cell cycle and induces EMT by upregulating Snail, Slug, and E‐cadherin promoters [90, 91]. CatL‐induced CUX1 activation may also contribute to triple‐negative breast cancer via estrogen receptor‐α repression [92]. Additionally, nuclear CatL involves CDP/Cux‐independent mechanisms of tumor promotion. In triple‐negative breast cancer, loss of BRCA1 activates nuclear CatL‐mediated p53‐binding protein 1 degradation, which acts as a replacement of BRCA1 that bypasses growth arrest and increases the survival of tumor cells. Moreover, this process activates DNA repair, which leads to increased therapy resistance [93]. The highly related CatL analogue, Cat V (CatV), also localizes to the nucleus in tumor cells, triggering hyperproliferation [94]. In breast cancer, nuclear CatV suppresses the expression of GATA3, a member of the zinc finger transcription factor family, by facilitating its turnover via proteasomes [95].

Cathepsin S (CatS) contributes to cancer progression by mediating autophagy. In human glioblastoma cells, CatS inhibition induces autophagy and an intrinsic pathway of apoptosis due to the production of reactive oxygen species [96, 97, 98] and subsequent suppression of PI3K/AKT/mTOR signaling and activation of JNK signaling [97]. Furthermore, CatS inhibition could also induce autophagy by activating the EGF receptor‐related ERK/MAPK signaling pathway [99].

Cathepsin K (CatK) is mainly implicated in bone matrix resorption; however, it modulates cancer signaling by regulating the TLR and Notch pathways [100, 101]. Additionally, CatK may regulate cytokines that are relevant for cellular crosstalk [102, 103, 104]. Furthermore, CatC promotes proliferation and metastasis in hepatocellular carcinoma by interacting with the tumor necrosis factor α (TNF‐α)/p38 MAPK signaling pathway [105].

CatX also interferes with key signaling pathways, facilitating IGF signaling and affecting downstream signaling through focal adhesion kinase (FAK) [106]. CatX is also involved in the MAPK/ERK and PI3K/Akt signaling pathways [68]. Additionally, multiple studies demonstrated that CatX importantly contributes to tumor cell signal transduction due to its interaction with integrin receptors [107]. Through integrin‐mediated pathways, CatX interacts with the FAK/Src signaling pathway and dysregulates cell migration [108].

Legumain promotes tumor progression via the PI3K/Akt signaling pathway [16] and cleaves tumor suppressor p53 in glioblastoma cells [109]. Additionally, inhibition of the p53 pathway and activation of the MYC pathway by legumain secreted from glioblastoma cells lead to the malignant transformation of normal astrocytes, which enhances the invasive ability of glioblastoma cells [110]. Tumor‐derived legumain interacts with endothelial integrin αvβ3 through its Arg‐Gly‐Asp (RGD) motif and indirectly downregulates the expression of zonula occludens 1 via the STAT3 signaling pathway, which can promote tumor metastasis by increasing the permeability of endothelial barriers [111, 112]. Moreover, in ovarian carcinoma cells, legumain interacts with integrin α5β1 and forms complexes that are secreted. These complexes are internalized by peritoneal mesothelial cells, in which they promote proliferation and migration via the FAK/Akt/ERK signaling pathways and contribute to EMT [113].

Both the proform and mature forms of CatD enhance cancer progression through both proteolytically dependent and independent manners [114]. Pro‐CatD acts as a protein ligand and stimulates the proliferation of breast cancer cells through an autocrine mechanism. It interacts with the IGF‐II receptor and can bind to the M6P/IGF‐II receptor on the surface of breast cancer cells [115]. Both CatD and pro‐CatD also promote cancer proliferation and migration by inducing phosphorylation of ERK and PI3K/Akt through a nonproteolytic mechanism [116, 117]. Moreover, it induces the outgrowth of fibroblasts by binding to the receptor of the low‐density lipoprotein receptor‐related protein 1, thus inhibiting intermembrane proteolysis that is regulated by this protein [118].

CatG interacts with oncogenic proteins from the TGF‐β pathway. It promotes TGF‐β signaling at the tumor–bone interface by proteolytic activation of matrix metallopeptidase 9 (MMP9), thus promoting tumor growth and enhancing osteoclast activation and subsequent bone resorption [119]. Furthermore, CatG also triggers the IGF‐I signaling pathway, which is partly responsible for cell aggregation [120]. Recently, it was demonstrated that CatG induces continuous phosphorylation of IGF‐1R and Akt, indicating that CatG‐specific IGF‐I increases are caused by digestion of the IGF‐binding protein IGFBP‐2, and not IGF‐I [121].

Lysosomal cathepsins also participate in regulating apoptosis [122, 123]. Cathepsins B, L, S, H, and K, but not cathepsins C and X, act proapoptotically by activating Bid to tBid, which activates caspases [124], and antiapoptotically by cleaving and inactivating members of the Bcl‐2 family [124, 125, 126, 127]. Moreover, CatB suppresses alternative forms of cell death by cleaving the lipid signaling enzyme sphingosine kinase 1 [128] and degrading receptor‐interacting Ser/Thr protein kinase 1 (RIPK1), consequently enforcing apoptosis [69, 129]. Recently, CatB was identified as an executor of ferroptosis, a necrotic form of cell death caused by inactivation of the glutathione system and uncontrolled iron‐mediated lipid peroxidation. [130]. By inhibiting complement‐mediated tumor cell death, CatL promotes tumor growth and survival of melanoma cells [131]. Next, inhibition of CatS was suggested to upregulate the expression of the proapoptotic Bim protein at post‐translation levels independent of the AMP‐activated protein kinase and MAPK signaling pathways [132]. In addition to CatB, CatS also degrades RIPK1 and suppresses necroptosis [69, 129].

In combination with other cathepsins, CatC is also involved in autophagic turnover by inducing endoplasmic reticulum stress and apoptosis mediated by JNK signaling [133]. By interacting with ribosomal protein P0 in the cytoplasm, CatX dysregulates apoptotic signaling and promotes tumor progression, as the knockdown of CatX led to G1 cell cycle arrest and apoptosis [134].

An important role in regulating apoptosis has also been demonstrated for CatD. It acts proapoptotically by activating Bax (directly or indirectly) [70, 114] and caspase‐8 [135]. Conversely, CatD can function as an antiapoptotic mediator of autophagy to protect cells under stress [136]. Recently, CatD inhibition was demonstrated to enhance the radiosensitivity of glioblastoma cells by attenuating autophagy through its effects on the fusion of autophagosomes and lysosomes [137]. In apoptosis, CatG downregulates survivin expression at the post‐translational level via 5‐lipoxygenase‐mediated reactive oxygen species production and significantly blocks TNF‐related apoptosis‐inducing ligand‐induced apoptosis [138].

### Lysosomal peptidases in invasion, migration, and metastasis

In general, lysosomal peptidases can promote tumor invasion, cell migration, and metastases directly by degrading ECM proteins or indirectly by cleaving other factors within proteolytic cascades (Table 1; reviewed in [16, 70, 139, 140]). The best studied lysosomal peptidase involved in these processes is CatB. It contributes to tumor progression by directly degrading ECM proteins such as laminin, fibronectin, collagen types I and IV, and proteoglycans. Additionally, CatB indirectly participates in degrading ECM by activating other peptidases that degrade ECM (reviewed in [56]). CatB also promotes cell migration by activating TLR 3 [141].

**Fig. 1:**
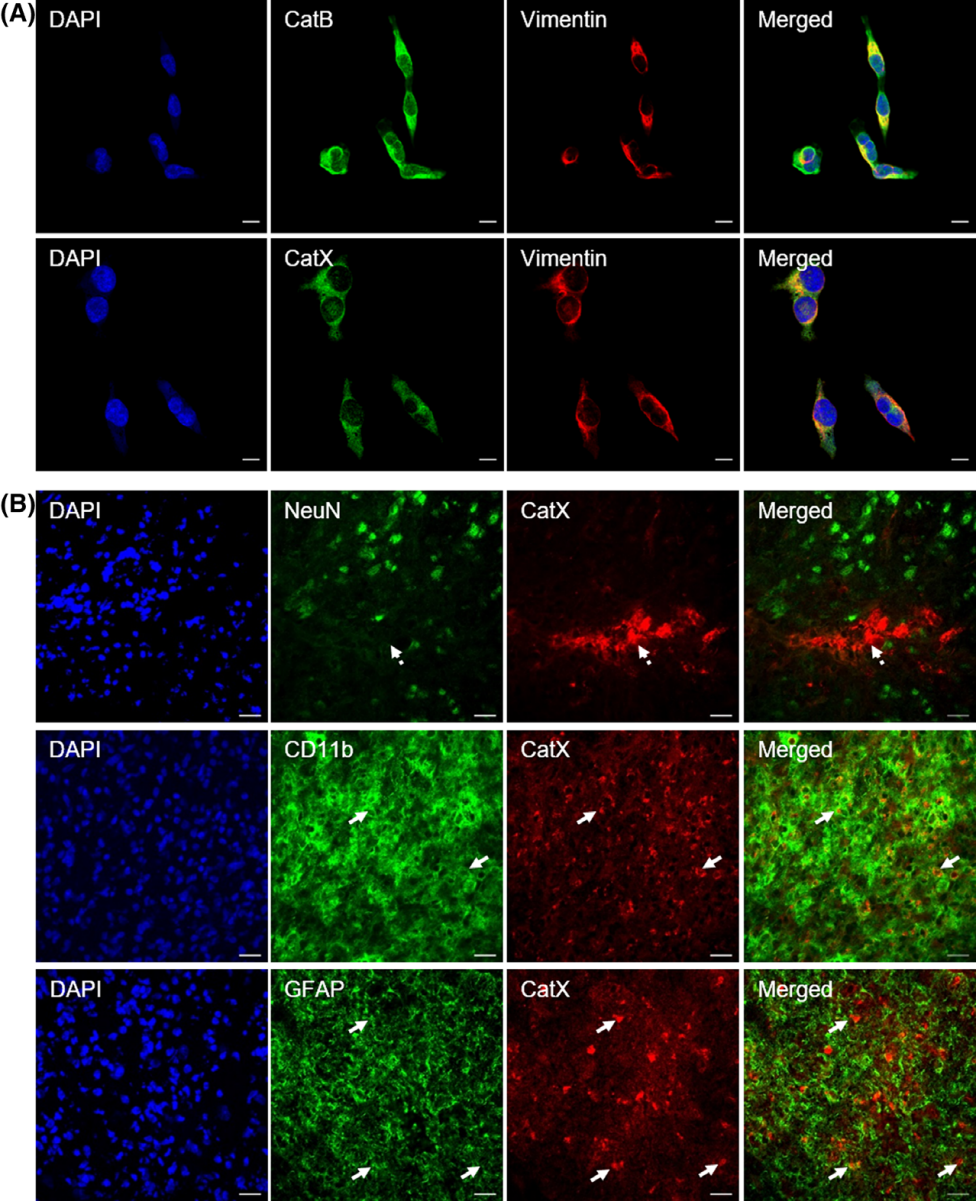
Cysteine Cat expression in tumor and brain cells. (A) Expression of CatB and X (CatX; green fluorescence) in the triple‐negative breast cancer cell line MDA‐MB‐231 that expresses high levels of the mesenchymal marker vimentin (red fluorescence). Scale bars, 10 µm. (B) Cell‐specific localization of CatX (red fluorescence) in the ipsilateral striatum of rat brain at 4 weeks after lipopolysaccharide injection, using cell‐type markers (green fluorescence) for neurons (NeuN), microglial cells (Cd11b), and astrocytes (GFAP). In the lesioned striatum, CatX was predominantly restricted to CD11b‐ and GFAP‐positive cells (white arrows), whereas neuronal cells were not positive for upregulated CatX (dashed arrow). Nuclei were counterstained with DAPI (blue fluorescence). Images were taken with an LSM 710 Carl Zeiss (Jena, Germany) confocal microscope, using zen imaging software. Scale bars, 20 µm.

Furthermore, CatB promotes tumor progression by inducing EMT in which higher CatB protein levels are linked with a more invasive mesenchymal cell phenotype (Fig. 1A) [142] and with EMT activators through the E‐box element in the CatB promoter [143]. E‐cadherin, a cell membrane protein and a component of adherens junctions, whose inactivation is a key event during EMT [144], has also been identified as a substrate of CatB [145].

Similar to CatB, CatL directly degrades proteins of the ECM and basal membrane (e.g., laminin, fibronectin, collagen types I and IV, and elastin) or activates other peptidases in proteolytic cascades (reviewed in [90, 146]). Moreover, extracellular CatL promotes tumor cell invasion via EMT by degrading E‐cadherin and other adhesion proteins [145]. Downregulation of CatL inhibits TGF‐β‐induced EMT and cancer cell invasion and migration [147]. It suppresses EMT‐inducing transcription factor Snail, which is associated with the PI3K/Akt and Wnt signaling pathways [148, 149]. CatL‐induced EMT through the Akt/glycogen synthase kinase‐3β/Snail pathway was demonstrated in glioma cells [149]. Several studies demonstrated that the regulatory effects of CatL on the EMT are attributed to the proteolytic processing of the transcription factor CUX1 [148, 149]. CatL induced by transcription factors (e.g., forkhead box O3A or K‐ras) or ionizing radiation was shown to play a crucial role in EMT [148, 150]. CatL is also involved in EMT by regulating RhoA and CDC42 signaling *in vitro* and *in vivo* [151].

Another Cat involved in EMT is CatV, as it increases levels of activated urokinase‐type plasminogen activator and alters the expression of proteins associated with EMT [152]. Among human cathepsins, CatV has the most potent elastolytic activity and is particularly important in intracellular elastin degradation in macrophages [153]. Additionally, CatS contributes to the degradation of ECM [154]. Its preinvasive function could be explained by its ability to cleave cell adhesion proteins, including E‐cadherin [145] and junctional adhesion molecule B [155]. Inhibition of CatS can reverse TGF‐β‐induced EMT, restore TGF‐β‐induced tight junction protein turnover, and consequently decrease the mobility of glioblastoma cells [156]. Furthermore, CatH regulates the migration of prostate cancer cells by processing talin (which affects integrin activation and adhesion) [157]. Additionally, CatK, which is predominantly extracellularly located, contributes to tumor progression by degrading collagen types I and II, elastin, vascular endothelial factor (VEGF), adiponectin, aggrecan, and osteonectin. Moreover, CatK is the Cat with the most efficient collagenase activity (reviewed in [146, 158]). In connection with other peptidases (e.g., MMPs), CatK cleaves stromal cell‐derived factor 1α, osteopontin, and stem cell factor, thereby altering cell signaling and enabling the release of stem cells into the ECM [158, 159].

Carboxymonopeptidase CatX contributes to tumor progression through mechanisms other than ECM degradation. Multiple substrates of CatX have been identified, including the β‐chain of integrin receptors, γ‐enolase, chemokine CXCL‐12, bradykinin, kallidin, huntingtin, and profilin‐1 (reviewed in [107]). CatX enhances migratory and invasive properties of tumor cells by interacting with integrin receptors and profilin‐1 [160, 161, 162] and promotes tumor progression by compensating CatB proteolytic activity [163, 164], bypassing senescence [165], and inducing EMT [142, 166]. In EMT, higher CatX levels correlate with an increased expression of mesenchymal markers (Fig. 1A) and decreased expression of epithelial markers. Overexpression of CatX during EMT is also associated with upregulation of MMP2, MMP3, and MMP9, which enable the remodeling of the ECM [142, 166].

In addition to its intracellular activity, legumain can be secreted into the tumor microenvironment (TME) in which it contributes to degrading and remodeling the ECM [16, 167], either by producing the mature forms of MMP2 and MMP9 [16, 168] or by processing cathepsins [169]. In gastric carcinoma, legumain knockdown resulted in changes in downstream EMT signaling pathways (e.g., Twist), with increased E‐cadherin and decreased mesenchymal markers [170]. CatD promotes tumor invasion, migration, and metastasis by cleaving ECM proteins, cytokines, and chemokines locally or by nonproteolytic mechanisms [114]. By activating CatB, CatD is capable of triggering processes downstream of the proteolytic cascades [75]. Next, proteolytically active CatD stimulates the activity of secreted plasminogen activators by degrading plasminogen activator inhibitor‐1 [171]. CatD was also observed to cleave endogenous inhibitors of cysteine peptidases: stefin B and cystatin C [172, 173]. Moreover, a recent study demonstrated the involvement of CatD and its proform in the migration of mesenchymal stem cells to tumor sites [174].

In contrast to most other cathepsins, higher CatE levels are associated with improved survival of cancer patients (reviewed in [70]). Deletion of CatE in mice resulted in the spontaneous development of mammary tumors and was associated with EMT and activation of the β‐catenin pathway [175]. CatE induces growth arrest and apoptosis in human prostate carcinoma tumor cell lines without affecting normal cells by catalyzing the proteolytic release of soluble TNF‐related apoptosis‐inducing ligand from the cell surface [176]. In addition, CatE was demonstrated to enhance the sensitivity of tumor cells to antitumor drugs [177].

The neutrophil peptidase CatG increases cell motility by proteolytic cleavage of cell surface proteins. It induces the E‐cadherin‐dependent aggregation of MCF‐7 cells [178]. E‐cadherin‐based cell–cell junctions are regulated by CatG promotion of E‐cadherin/catenin and E‐cadherin/protein kinase D1 complex formation and Rap1 activation in MCF‐7 cells [179]. CatG also activates proteinase‐activated receptor 4 that triggers cell membrane blebbing, a mechanism recognized as an important regulator of cell migration, cancer cell invasion, and vesicular content release [180].

Tumor angiogenesis is another important mechanism during tumor progression. The hypoxic TME activates several signaling molecules, including VEGF, platelet‐derived growth factor, interleukins (ILs), and TGF‐β, which all promote the proliferation of endothelial cells. Proteolysis importantly contributes to angiogenesis, as it enables the migration and invasion of endothelial cells via ECM degradation, regulates the activity of cytokines and growth factors important for angiogenesis, and releases pro‐ and antiangiogenic factors [69, 181].

In addition to the promotion of angiogenesis by degrading ECM [146], CatB enhances angiogenesis by degrading matrix‐associated angiogenesis inhibitors, such as the endogenous tissue inhibitors of metalloproteases TIMP‐1 and TIMP‐2 [182]. Additionally, by degrading the ECM, CatB also releases growth factors bound to ECM proteins such as VEGF and TGF‐β [75]. Next, CatL promotes invasion and integration of circulating endothelial progenitor cells into ischemic tissue that is required for the formation of new blood vessels [183] and that contributes to angiogenesis by releasing growth factors from the ECM (reviewed in [90]). In human gastric cancer, CatL also contributes to angiogenesis by regulating the CDP/Cux/VEGF‐D pathway [84].

CatS generates the antiangiogenic peptides canstatin and arrestin by cleaving collagen type IV and proangiogenic γ2 fragments by cleaving laminin [184]. CatS has also been suggested to interact with VEGF during angiogenesis, [154]. In the establishment and functional development of tumor vasculature, important roles were also recognized for CatH [185] and CatK [70, 146]. Pro‐CatD and mature CatD also possess proangiogenic activity [114, 186] and have been suggested to cleave and release proangiogenic basic fibroblast growth factor from the ECM [187] and to activate VEGF [188]. The proangiogenic role of CatD was further demonstrated by its activation of MAPK and PI3K/Akt signaling via a nonproteolytic mechanism present at higher nonacidic pH in the pre‐TME [114, 116]. Conversely, CatD is involved in the degradation of antiangiogenic factors, such as angiostatin, prolactin, and endostatin [70, 114]. Furthermore, CatE inhibits angiogenesis by upregulating the antiangiogenic mediators IL‐12 and endostatin [189]. Finally, CatG upregulation in cancer cells promotes tumor vascularization via upregulation of TGF‐β signaling, VEGF, and monocyte chemotactic protein 1 [190].

### The role of lysosomal peptidases in immune escape mechanisms in cancer

Eliminating cancer cells is the ultimate goal of the immune response during cancer immunosurveillance and immunotherapy. CTLs and NK cells are the key effectors in this process. CTL activation is an antigen‐specific process requiring specific antigen recognition, activation, and differentiation into effector CTLs, whereas NK cells exist in a preactivated state and can rapidly and effectively kill tumor cells that have downregulated major histocompatibility complex class I molecules (reviewed in detail in [191]). Furthermore, whereas CTLs kill differentiated tumor cells, NK cells also have the ability to kill stem‐like tumor cells [192, 193]. Both CTLs and NK cells deploy the same killing mechanisms, through either the death receptor pathway or cytotoxic granule release [194]. Cytotoxic granules contain proforms of perforin and several peptidases, including granzymes (granzymes A, B, H, M, and K in humans) [195]. Perforin is a calcium‐dependent pore‐forming protein that requires proteolytic removal of 20 amino acids at its C terminus for liberation of its C2 domain and activation. Perforin release and binding to the cell membrane is required for granzyme entry and apoptosis induction in target cells [196]. CatL has been implicated in the C‐terminal processing and activation of perforin, as the selective inhibition of CatL reduced perforin activation and the killing capacity of human NK cell lines and primary mouse CTLs. However, *in vivo*, CatL deficiency reduced the amount of active perforin but did not affect the overall cytotoxicity of NK cells in mice [197]. Granzymes are serine peptidases that are stored in cytotoxic granules as inactive precursors that require the removal of the N‐terminal dipeptide for their activation [198]. Even though CatC has an essential role in the *in vivo* activation of granzymes A and B, residual granzyme B activity is sufficient to combat viral infection in CatC^−/−^ mice [199]. Furthermore, CatH has been identified as an additional progranzyme convertase [200].

The endogenous inhibitor cystatin F (CysF), a member of the type II cystatin family, predominantly acts on peptidases located within the endo/lysosomal system, including cytotoxic granules. The molecular form of CysF governs its inhibitory profile. After synthesis, CysF forms disulfide‐linked dimers that do not inhibit the C1 family of cysteine peptidases but strongly inhibit legumain through a distant, second binding site [201]. N‐terminal cleavage after CysF translocation to endo/lysosomes [202] produces active monomeric CysF that is a strong inhibitor of cathepsins C, H, and L [203, 204]. Additionally, secreted CysF can be internalized, transported to endo/lysosomes, and, as such, can regulate cysteine peptidase activity *in trans* [49, 205].

In NK cells, CysF was shown to reduce granule‐mediated cytotoxicity by regulating the activity of the main granzyme convertases, cathepsins C and H [49]. Furthermore, increased CysF levels and decreased CatC and CatH levels are associated with target‐induced inactivation of NK cytotoxicity, referred to as ‘split anergy’ [206]. Split anergy of NK cells can be triggered through interaction with tumor cells and monocytes and is characterized by high cytokine secretion and reduced efficacy in killing target cells [206]. Increased CysF levels were also detected in anergic CTLs [207]. Recently, CysF was also found in CD4^+^ T cells that acquired cytotoxic functions during long‐term cultivation [208]. In contrast to most other type II cystatins, which are generally downregulated in tumors [62], CysF was found to be markedly upregulated in several types of cancer. In colorectal tumors, high CysF mRNA levels were shown to correlate with an increased risk of liver metastasis and poor survival [209, 210]. Furthermore, CysF gene expression was shown to be higher in glioblastoma tissues than in normal brain tissues, and CysF mRNA levels were shown to correlate with shorter patient survival [211, 212]. Finally, CysF was found to be expressed in patient‐derived glioblastoma stem‐like cells [211]. Recently, it was shown that extracellular CysF attenuates granzyme‐mediated cytotoxicity in CTLs [213] and decreases the susceptibility of a glioblastoma cell line to NK cytotoxicity [211]. Apart from the effects on properforin and granzyme activation, increased extracellular CysF levels can affect the activity of immune cells through several additional mechanisms.

CatC^−/−^ mice exhibit reduced expression of the β2 integrin receptors CD11c and CD11b on CTLs and CD11c on dendritic cells. These β2 integrin receptors are adhesion and signaling molecules that are critically important for cell‐to‐cell contact and leukocyte recruitment to inflammation sites [214]. Furthermore, apart from its role in activating perforin, CatL has been implicated in regulating the cytotoxic efficacy of CTLs by cleaving complement C3; namely, upon activation of T‐cell receptor, CatL cleaves complement component C3 into C3a and C3b fragments, which in turn engage and activate their corresponding receptors (C3aR and CD46). Signaling through CD46 is necessary for optimal CTL cytotoxic activity [215]. Engagement of C3aR and CD46 is also important for the optimal survival and differentiation of CD4^+^ T lymphocytes toward the Th1 phenotype [216]. In CD4^+^ lymphocytes, CD46 costimulation also induces the expression of legumain, which processes single‐chain CatL into its active two‐chain form in human CD4^+^ T lymphocytes [217]. Inhibition of legumain activity in human CD4^+^ T lymphocytes reduces the generation of the CatL active forms and C3a and induction of IFN‐γ‐secreting cells by approximately 50% [217].

Conventional CD4^+^ lymphocytes cannot kill cancer cells directly; however, by secreting various cytokines, they play a significant role in shaping antitumor immune responses. A subset of Th17 helper lymphocytes plays an important role in cancer‐related inflammation, which can be unfavorable or beneficial, depending on the setting and cancer type [218]. Both CatL and CatS have been implicated in the differentiation of the Th17 subset. CatL is an intrinsic promoter of Th17 development in CD4^+^ cells [219], and cell differentiation can be blocked by specific exogenous CatL inhibitors [220]. In mice, conventional CD4^+^ cells more readily differentiate to the Th17 cell type when lacking an endogenous CatL inhibitor, serpin B1 [220]. Through activation of the protease‐activated receptor 2 receptor on dendritic cells, which drives IL‐6 production and secretion, CatS has been implicated in the generation and expansion of Th17 lymphocytes [221].

Regulatory T cells are key factors in tumor immune escape, as they can inhibit the activation and differentiation of CD4^+^ helper T cells and CTLs to induce reactivity against tumor‐expressed antigens through a variety of mechanisms [222]. It was shown that CatS inhibition enhances the immunosuppressive activity of regulatory T cells under normal conditions, whereas, in the presence of tumor cells, CatS inhibits regulatory T cells and stimulates antitumor immunity by promoting CTL proliferation and survival [223]. Similar observations have been made in CatK^−/−^ mice, in which regulatory T cells were potent suppressors of effector T lymphocytes under normal conditions [224].

Important components of the TME are tumor‐infiltrating immune cells of myeloid origin, which are actively involved in bidirectional interactions with tumor cells. Tumor‐associated macrophages (TAMs) constitute the major leukocyte population in tumors [225]. TAMs are generally categorized into classically activated M1 macrophages (which typically exert antitumor functions) and alternatively activated M2 macrophages (which can inhibit T‐cell‐mediated antitumor immune responses, promote tumor angiogenesis, and lead to tumor progression) [226]. In addition to converting macrophages toward the tumor‐promoting phenotype [226], the TME drives the expansion of a heterogenic immature population of myeloid‐derived suppressor cells (MDSCs). MDSCs have a profound effect on the course of the antitumor immune response and on the effector functions of adaptive immune cells [227]. MDSCs and TAMs also support cancer progression by enhancing cancer cell stemness [228, 229] and resistance to chemotherapy [230], releasing proangiogenic peptides necessary for angiogenesis [231], facilitating tumor intravasation into the circulation, and contributing to tumor metastasis.

Increased proteolytic activity of cysteine cathepsins has also been associated with the tumor‐promoting roles of MDSCs [163, 232]. Recently, it was shown that during MDSC differentiation, the overall levels of cysteine cathepsins increase, with the most pronounced increase in the activities of CatL and CatX. Blocking their activity with small‐molecule inhibitors (CLIK‐148 and Z9 for CatL and CatX, respectively) in tumor and immune cell cocultures showed that CLIK‐148 significantly increases CD8^+^ cytotoxicity [233]. Proteomic analysis of MDSCs from metastatic tumors revealed decreased neutrophilic granule protein compared with that of those from nonmetastatic counterparts. Neutrophilic granule protein is structurally similar to type II cystatins and can inhibit CatB and consequently reduce tumor vascularization, growth, and metastasis [234].

In a mouse model of hereditary polyposis, increased Cat activity was detected in macrophages and MDSCs, which were infiltrating the lesions. MDSCs were shown to depend on CatB activity, since CatB^−/−^ mice failed to accumulate MDSCs [235]. Furthermore, treatment with anti‐TNF‐α antibodies reduced MDSC density and decreased the detectable activity of polyp‐specific CatB in the same mouse model [236]. This is in line with a previous study showing that CatB deficiency abrogated the trafficking of TNF‐α‐containing vesicles to the cell membrane in different types of monocytic cells [237].

A mouse SCID‐hu model of bone metastasis revealed that stromal‐derived CatK may be an important factor in the colonization and growth of tumors in the skeleton. CatK was suggested to interfere with macrophage‐regulated inflammatory processes in bones [238]. A further study on a model of bone metastasis in CatK^−/−^ mice showed critical involvement of bone marrow macrophage‐derived CatK in bone tumor progression [239]. In this model, macrophage infiltration was reduced in the absence of CatK and correlated with lower inflammation levels [239]. Bone marrow macrophage‐derived CatK was suggested to be involved in pathways that are driven by chemokine (C–C motif) ligand 2 and cyclooxygenase 2 and contribute to tumor progression and bone metastasis. Increased cyclooxygenase 2 activity, first associated with inflammation, is also frequently increased within the TME. This leads to increased synthesis of eicosanoid prostaglandin 2, which is a driver of the functional differentiation of TAMs and MDSCs [240, 241]. Furthermore, it was shown that cathepsins are involved in post‐translational cyclooxygenase 2 maturation and catalytic regulation, as their inhibition with the broad‐spectrum Cat inhibitors E64d and ALLn was shown to block cyclooxygenase 2 maturation, resulting in diminished prostaglandin 2 formation [242]. Furthermore, CatK induced the overexpression of CatB, another important driver of tumor progression [239].

Macrophage‐derived CatX was found to facilitate cancer cell invasion through the Arg‐Gly‐Asp (RGD) motif in its prodomain, which regulates interactions with integrins and the ECM [235]. Genetic ablation of CatS leads to the depletion of several proinflammatory chemokines, most notably the chemokine (C‐C motif) ligand 2, which is required for the recruitment of MDSCs and TAMs. This regulation is transcriptionally mediated. CD74 (also known as the major histocompatibility complex II chaperone invariant chain) is cleaved by CatS in endosomes, resulting in the release and nuclear translocation of its intracellular domain and the activation of transcription factor NF‐κB, which transcriptionally regulates chemokine (C‐C motif) ligand 2 expression [243].

Chemotherapy‐induced MDSC depletion is generally favorable in tumor therapy; however, it was shown that cysteine cathepsins play an important role in some unfavorable off‐target effects of chemotherapy. It was shown that 5‐fluorouracil and gemcitabine, which selectively target and kill MDSCs, indirectly induce lysosomal membrane permeabilization and CatB leakage into the cytoplasm. Upon lysosomal membrane permeabilization, CatB was shown to directly interact with the leucine‐rich repeat domain of NLRP3 and activate the inflammasome, the multiprotein platform for caspase‐1 activation, which is necessary for conversion of pro‐IL‐1β into mature IL‐1β. This leads to IL‐1β secretion, which stimulates CD4^+^ T lymphocytes to produce IL‐17, potentially leading to angiogenesis and subsequent tumor relapse [244]. Similarly, the commonly used chemotherapeutic paclitaxel was shown to increase TAM infiltration into the tumor site, which contributes to increased Cat activity within the TME. An *in vitro* study showed that macrophage‐derived CatS and CatB, but not CatC and CatL, protect tumor cells against cell death induced by paclitaxel, etoposide, and doxorubicin [245].

## Lysosomal peptidases in neurodegeneration

Neurodegeneration refers to the progressive loss of neuronal structure or function and can lead to devastating neurological conditions, such as Parkinson's disease (PD), AD, and ALS. Impaired endo/lysosomal systems have been linked to the pathogenesis of neurodegenerative diseases and disrupted cellular homeostasis, thus contributing to neurodegeneration [246].

### Lysosomal peptidases in brain pathologies related to misfolded proteins

Misfolded proteins that cause neurodegeneration are generated over the course of aging by post‐translational modifications of native proteins or genetic mutations of otherwise nonpathogenic proteins [247]. In several neurodegenerative diseases, specific proteins begin to aggregate in individual brain regions at early, commonly nonsymptomatic stages of the disease, whereas additional brain regions become involved in the advanced stages of the disease [248]. Misfolding and aggregation of amyloid beta (Aβ) protein in senile plaques and tau protein in neurofibrillary tangles represent the most widely accepted pathogenic markers of AD [249, 250]. However, another early feature of AD is lysosomal dysfunction, and accruing evidence suggests that lysosomal peptidases may be key pathogenic players (Table 2) [251, 252].

**Table 2:** Significance of lysosomal peptidases in neurodegeneration.

Type of Cat	Function	Pathogenesis	References
CatD	Proteolytic cleavage of Aβ and tau protein	AD	[253, 254, 255, 256, 257]
	Proteolysis of apoE into toxic peptide	AD	[263]
	Proteolysis of α‐syn; disturbance in CatD function leading to pathogenesis	PD	[303, 305, 306]
	Involved in 6‐OHDA‐induced apoptosis of dopaminergic cells	PD	[312]
	Mutations in CatD gene	NCL type 10	[363]
CatE	Proteolysis of apoE into toxic peptide	Aging, AD	[263]
CatB	β‐secretase activity in APP cleavage into toxic Aβ peptide; preference for cleaving wild‐type β‐secretase substrate	AD	[271, 272, 273, 274]
	Proteolysis of α‐syn; formation of intracellular α‐syn aggregates	PD	[306, 309]
	Involved in motor neuron degeneration	ALS	[317]
	Proteolytic degradation of mitochondrial transcription factor A	Neuroinflammation, aging	[331]
	Involved in caspase‐1 activation leading to secretion of interleukin‐1β; involved in caspase‐11 activation	Neuroinflammation	[343, 344]
	Loss of CatB activity leads to accumulation of free cholesterol in late endo/lysosomes	NPC	[371, 372]
CatL	β‐secretase activity in APP cleavage into toxic Aβ peptide	AD	[271]
	Proteolysis of α‐syn	PD	[306]
	Involved in 6‐OHDA‐induced apoptosis of dopaminergic cells	PD	[313]
	Contributes to inflammatory responses when released from activated microglia	Neuroinflammation	[337]
	Loss of CatL activity leads to accumulation of free cholesterol in late endo/lysosomes	NPC	[372]
CatS	β‐secretase activity in APP cleavage into toxic Aβ peptide	AD	[271]
	Degrades monomers and dimers of the Aβ peptide and APP *in vitro*	AD	[289]
CatC	Involved in chemokine production	Neuroinflammation	[345]
	Promotes M1 microglia polarization via the Ca^2+^‐dependent PKC/p38MAPK/NF‐κB pathway	Neuroinflammation	[346]
CatF	Mutations in CatF gene	NCL type 13	[365, 366, 367, 368, 369]
	Accumulation of eosinophilic granules and lipofuscin in neurons is increased in association with decreased CatF expression.	NCL type 13	[364]
CatX	Proteolytic cleavage of the C‐terminal end of γ‐enolase, abolishing its neurotrophic activity	Aging, AD	[294, 295, 296]
	Involved in 6‐OHDA‐induced apoptosis of dopaminergic cells	PD	[314, 315]
	Contributes to inflammatory responses when released from activated microglia	Neuroinflammation	[349, 352]
Legumain	Phosphorylation of tau protein; degradation of tau protein	Aging, AD	[298, 299]

Aspartyl peptidase CatD degrades both Aβ [253, 254, 255] and tau [256, 257] and is strongly implicated in the pathogenesis of AD [258]. In AD patients, CatD levels are high in cortical and hippocampal neurons [259], amyloid plaques, and cerebrospinal fluid [260, 261, 262]. It has been suggested that CatD is also involved in the proteolysis of both lipid‐free recombinant full‐length human apolipoprotein E (apoE) and lipidated human plasma full‐length apoE4 into toxic peptide, contributing to the progression of AD [263]. Additionally, another aspartyl peptidase, CatE, processes lipid‐free recombinant human apoE to a much greater extent than lipidated apoE [263] and appears to be involved in neurodegeneration associated with brain ischemia and aging [264, 265]. CatE is present in senile plaques in AD brains [266] and exhibits increased expression and lysosomal localization in cortical and brainstem neurons of aged rats [264].

Cysteine cathepsins are also associated with neurodegeneration (Table 2) [14, 252]. Among them, CatB and CatL might be crucial in intracellular catabolism related to age‐associated changes that lead to neuronal death [265, 267]. High CatB and CatL levels were found in neurons and amyloid plaques in AD brain [268]. Conversely, mice lacking CatB and CatL exhibited atrophy in cerebral and cerebellar brain regions, suggesting the necessity of these cathepsins for neuronal development [269]. Furthermore, suppression of CatB and CatL by exposing cultured hippocampal slices to a selective Cat inhibitor provoked changes similar to those occurring during brain aging, for example, an increased number of lysosomes and the formation of neurites [270]. Nevertheless, the cysteine cathepsins B, L, and S were identified as enzymes possessing β‐secretase activity for the cleavage of amyloid precursor protein (APP) into toxic Aβ peptide [271]. Among them, CatB in secretory vesicles is most strongly defined as a β‐secretase for the production of the neurotoxic Aβ peptide in AD [272, 273, 274].

CatB shows a clear preference for cleaving wild‐type β‐secretase substrate, whereas it shows essentially no activity for Swedish mutant β‐secretase substrate [271, 274]. Inhibition by the cysteine peptidase inhibitor E64d and related inhibitor CA‐074Me (which preferentially inhibits intracellular CatB) reduces brain Aβ peptide levels and improves memory in an AD mouse model. Conversely, E64d has no effect in this model expressing the Swedish mutant β‐secretase site of APP [271, 274, 275, 276, 277]. Kindy et al. showed improved memory deficits after CatB gene knockout in an AD mouse model expressing the wild‐type β‐secretase site of APP that is present in most AD patients [278]. Conversely, CatB degraded Aβ via C‐terminal truncation, leaving its role in Aβ metabolism unclear [279, 280]. Mueller‐Steiner et al. demonstrated that CatB actually reduces Aβ peptide levels, especially the aggregation‐prone species Aβ1–42, through proteolytic cleavage. They suggested that inhibition or loss of CatB activity could interfere with the protective function of CatB and thus promote the development of AD [280]. A recent study by Oberstein et al. on cultured astrocytes showed varying roles of CatB in Aβ regulation that might depend on different cellular localizations of active CatB. Nonlysosomal CatB mediated Aβ production in astrocytes, while Aβ degradation depended on lysosomal CatB and the production of Aβ peptides; this highlights the importance of considering organelle targeting in drug development to promote Aβ degradation [281]. Nevertheless, elevated CatB levels have been detected in AD patient brains in membrane‐bound organelles, degenerating neuronal perikarya, reactive astrocytes, and extracellularly near neuritic plaques [268, 282, 283]. In addition, CatB plasma levels are elevated in AD patients [284, 285]. Therefore, CatB has been recognized as a crucial pathogenic factor and potential target in AD [286].

Another enzyme with β‐secretase activity that is associated with the pathogenesis of AD is CatS [271]. Transfection of human kidney cells with CatS increased Aβ secretion, whereas the Cat inhibitor E64d reduced this secretion [287]. CatS is weakly detected in normal human brain, whereas CatS immunoreactivity was observed in tangle‐bearing neurons, astrocytes, and rare senile plaques in AD brain [288]. In addition, Liuzzo et al. demonstrated that CatS can degrade Aβ peptide monomers and dimers *in vitro* [289]. It is known that Aβ peptides are taken up predominantly by microglia and are accumulated and degraded in microglial endo/lysosomal systems [290]. Thus, microglial CatS may assist in the extracellular clearance of intracellularly formed Aβ or soluble Aβ and modulate Aβ peptide levels at the very initial stages of peptide aggregation, which in turn may affect Aβ neurotoxicity [291]. Besides CatS, enhanced CatL and CatH levels were found in the majority of astroglia and microglia in the hippocampus of AD patients, both within and outside senile plaques [292, 293], indicating the pathogenic role of CatL and CatH in age‐related neurodegeneration.

Another lysosomal cysteine peptidase strongly linked to age‐related neurodegeneration is CatX. High levels and proteolytic activity of CatX have been observed in degenerating brain regions of transgenic AD mouse models and around senile plaques in AD patient brains [294, 295]. A transgenic AD mouse model revealed CatX upregulation in microglial cells surrounding amyloid plaques and CatX colocalization with its target γ‐enolase in the vicinity of the plaques [294, 295]. Furthermore, CatX contributes to Aβ‐related neurodegeneration through proteolytic cleavage of the C‐terminal dipeptide of γ‐enolase, abolishing its neurotrophic and neuroprotective activity [295]. Consequently, γ‐enolase cannot impair Aβ‐induced apoptosis through neurotrophin receptor p75^NTR^ signaling [296]. Furthermore, a comprehensive comparative gene expression analysis of mouse models of AD, multiple sclerosis, and stroke found that CatX is one of the eighteen genes whose expression is increased in all three models of central nervous system (CNS) disorders [297].

In addition, legumain, which is activated in aging and AD brains [298], is involved in tau phosphorylation by inactivating protein phosphatase 2 inhibitor I2 [299]. Legumain is also involved in tau degradation, thereby abolishing its microtubule assembly function and inducing its aggregation that leads to neurodegeneration [298].

The accumulation of misfolded proteins plays a central role in the pathogenesis of PD and impairs lysosomal function [300]. The crucial pathological event in PD involves the aggregation of alpha‐synuclein (α‐syn) from intermediate soluble oligomers to structurally complex and insoluble fibrils found in Lewy bodies and neurites [301]. The lysosomal degradation pathway is mostly responsible for the clearance of α‐syn oligomers, and disturbance in lysosomal function has been linked to the accumulation of α‐syn oligomers and α‐syn‐mediated cell death [302].

CatD was the first lysosomal peptidase found to protect against α‐syn aggregation and toxicity in mouse models [303, 304, 305]. *In vitro* and *in vivo* studies demonstrated that CatD mediates the lysosomal proteolysis of α‐syn under physiological conditions [303, 305, 306]. In agreement, overexpressed CatD was found to effectively degrade α‐syn in dopaminergic cells, whereas CatD‐deficient mice accumulated insoluble α‐syn in the brain, thereby facilitating α‐syn toxicity [303]. Similar results, that is, α‐syn accumulation in CatD‐deficient animals and neuroprotection against α‐syn toxicity in CatD‐overexpressing neuroglioma cells, were also observed by Qiao et al. [304]. Moreover, using CatD‐deficient lysosomes, CatD has been demonstrated to be the main lysosomal enzyme involved in α‐syn degradation [305]. Recently, damaging variants of CatD were found to be genetically linked to lysosomal dysfunction and PD pathology in a large screening of PD patients [307]. Furthermore, an additional study showed that a PD‐associated CatD variant (A239V) exhibited increased enzymatic activity accompanied by increased α‐syn levels [308].

Conversely, cysteine cathepsins have been shown to be essential in lysosomal degradation of α‐syn. Using lysosomal extracts and mass spectrometry analysis, CatD was found to only generate C‐terminal α‐syn fragments, whereas the majority of α‐syn degradation was associated with CatL, and to a lesser extent with CatB [306]. In a cell‐based study using the CatB inhibitor CA‐074Me and CatD inhibitor pepstatin, CatB, but not CatD, was found to be the major enzyme involved in fibril‐induced formation of intracellular α‐syn aggregates. Similar results were obtained using CatB knockdown [309]. Further studies are therefore needed to resolve this discrepancy.

### Lysosomal peptidases in progressive degeneration accompanied by neuronal loss

Another feature of PD is a progressive degeneration of the dopaminergic projection in the *substantia nigra compacta* (SNc), which results in loss of dopaminergic neurons in the SNc. The important role of certain cysteine cathepsins in neurodegenerative disorders is becoming well established in acute pathological conditions and chronic diseases with inflammatory pathologies such as PD (Table 2; reviewed in [65]). [310, 311]. The lysosomal proteolytic system participates in the apoptosis of neuronal‐like cells induced by 6‐hydroxydopamine (6‐OHDA), a common neurotoxin model of PD [312].

Increased CatB and CatD expression has been shown in a 6‐OHDA model of PD. Cells treated with pepstatin A, a CatD inhibitor, showed a significant decrease in cell death; however, CA‐074Me, a CatB inhibitor, failed to protect cells from 6‐OHDA‐induced cell death [312]. Also, other cysteine peptidases, for example, CatL and CatX, play a role in the apoptosis of dopaminergic neurons. CatL mediates 6‐OHDA‐induced apoptotic events leading to PD‐related neurodegeneration [313]. As such, reports have shown increased CatL expression in dopamine neurons in ipsilateral SNc of a rat PD model and in PD patients [310]. An *in vitro* study revealed that also CatX promotes 6‐OHDA‐induced apoptosis and subsequent neuronal toxicity, and CatX inhibition exerts potent neuroprotection of dopaminergic‐like neuronal cells, designating peptidases as pathogenic factors in the progressive loss of dopaminergic neurons [314]. Indeed, another *in vivo* study revealed CatX upregulation in a 6‐OHDA model of PD. 6‐OHDA injection into the medial forebrain bundle increased CatX expression and activity in the SNc at the ipsilateral side, with the simultaneous reduction in numerous dopaminergic nigrostriatal neurons. This prominent CatX upregulation was restricted to dopaminergic neuronal cells at early time points after the injection, whereas at late time points, CatX upregulation was restricted to glial cells concentrated in the ipsilateral SNc [315].

Another neurodegenerative disease where progressive neuronal loss is present is ALS. ALS is characterized by selective degeneration and death of motor neurons associated with the accumulation of misfolded proteins and insoluble inclusions [316]. At first, only CatB was found to be involved in motor neuron degeneration, whereas cathepsins H, L, and D were not significantly affected in ALS patients [317]. However, further studies showed that the expression of CatB, CatL, and particularly CatD increases in ALS spinal cord with a concomitant change in the distribution and lysosomal associations of CatD [318]. ALS model mice revealed that the expression and protein levels of cathepsins B, L, S, X, and D all increased in the spinal cord in ALS mice, generated by mutating the copper/zinc superoxide dismutase (*SOD1*) gene [294, 318, 319]. Additionally, a cDNA microarray analysis on postmortem spinal cord specimens of four sporadic ALS patients revealed major changes in mRNA expression of 60 genes, including increases in CatB and CatD [320]. Nevertheless, CatB‐knockout mice showed a lower rate of motor neuron death after nerve injury [321], suggesting that CatB inhibition is beneficial for motor neuron survival. It is therefore likely that lysosomal enzymes, such as cathepsins, are activated in the ALS spinal cord and may contribute to the disease [318].

### Microglial lysosomal peptidases promote neuroinflammation

Accumulating evidence suggests that chronic innate neuroinflammation mediated by microglia and astrocytes is involved in the progressive nature of neurodegenerative disorders [322]. During neuroinflammation, activated microglia and astrocytes release a variety of cytokines, chemokines, and toxic factors, which may lead to subsequent neuronal toxicity. This is accompanied by oxidative stress [323], mitochondrial dysfunction [324], and activation of the apoptotic cascade [325, 326], all of which lead to aggressive neuronal loss and exacerbate neurodegeneration [327, 328, 329, 330]. In addition to inflammatory molecules, activated microglia also secrete lysosomal peptidases, which support various immune functions [290, 331, 332]. Inflammatory stimuli such as lipopolysaccharide (LPS), which also induces death of nigral dopaminergic neurons through microglial activation, substantially increase microglial secretion of lysosomal peptidases [289, 333, 334, 335, 336]. In the microglia cell line BV2, LPS exposure leads to increased levels of the cysteine cathepsins B, K, S, and X in culture supernatants [336]. Substantially increased CatL secretion from microglia has been observed in response to LPS treatment for 1 h, which is earlier than the upregulation of proinflammatory cytokines, indicating that the earlier release of lysosomal CatL in microglia may contribute to inflammatory responses [337]. In addition, CatL inhibition alleviates the microglia‐mediated neuroinflammatory responses through caspase‐8 and NF‐κB pathways [338]. Furthermore, cathepsins B [339], L [338], H [340], C [341], and X [342] are upregulated in different brain regions following LPS‐induced neuroinflammation.

Microglial CatB has been extensively studied in neuroinflammation. Cytoplasmic CatB enhances the activation of caspase‐1, therefore promoting the microglial production and secretion of proinflammatory cytokine IL‐1β [343] through the pyrin domain‐containing protein 3 inflammasome‐independent processing of procaspase‐3 in phagolysosomes [344]. The leakage of CatB from the endo/lysosomal system during aging is associated with the proteolytic degradation of mitochondrial transcription factor A, which can stabilize mitochondrial DNA. Therefore, microglial CatB could function as a major driver of inflammatory brain diseases and brain aging (reviewed in [331]). Similarly, the expression of microglia‐secreted CatC is enhanced during CNS inflammation. CatC expression in the brain is induced predominantly in activated microglia [341], and CatC plays a role in promoting chemokine production in CNS inflammation [345]. CatC promotes microglia M1 polarization and aggravates neuroinflammation via the Ca^2+^‐dependent PKC/p38MAPK/NF‐κB pathway [346]. Similarly, the expression of microglia‐secreted CatS is increased during CNS inflammation and aging in mice [319]. Altered CatS expression is controlled by a built‐in molecular clock in cortical microglia; the circadian expression of CatS is involved in diurnal variations of synaptic strength via proteolytic modification. CatS has also been associated with some sleeping disorders, as its genetic ablation reduces synaptic strength during sleep by inducing hyperlocomotor activity that is required to obtain novel information after waking [347].

CatX has also been associated with inflammatory processes leading to neurodegeneration. It is disproportionately expressed and secreted by microglia and astrocytes in response to neuronal damage and inflammatory stimulus, both *in vitro* and *in vivo* [336, 348, 349, 350]. *In vitro*, the inflammatory stimulus LPS substantially increases CatX secretion from microglia, leading to neurodegeneration mediated by microglia activation [336, 349]. This was confirmed by the CatX‐specific inhibitor AMS36, which suppressed the production of proinflammatory molecules and attenuated cytokine release from activated microglial cells, leading to reduced microglia‐mediated neurotoxicity [349]. *In vivo*, unilateral LPS injection into the striatum increased CatX expression and activity in the striatum and surrounding areas on the ipsilateral side. This prominent CatX upregulation was restricted to activated microglia and reactive astrocytes (Fig. 1B). Moreover, administration of a CatX inhibitor along with LPS injection revealed the potentially protective role of such inhibitors in neuroinflammation‐induced striatal lesions [342]. Additionally, dendritic cells in the aging brains of mice have increased CatX protein levels, indicating on its role in neuroinflammation [351]. Allan et al. showed that CatX‐deficient mice have reduced neuroinflammation and decreased circulating IL‐1β levels during experimental autoimmune encephalomyelitis, a well‐known model of multiple sclerosis [352]. Multiple sclerosis is an autoimmune disease characterized by immune‐mediated inflammation, which attacks the myelin sheath. Hypomethylation of the CatX locus has been proposed as an epigenetic risk factor for multiple sclerosis [353].

Several observations suggest that also other cysteine cathepsins play a role in immune‐mediated inflammation involved in multiple sclerosis. Markedly increased levels of CatB and CatS in peripheral blood mononuclear cells, serum, and cerebrospinal fluid of multiple sclerosis patients have been determined [354, 355, 356, 357] and confirmed in an experimental models of autoimmune encephalomyelitis [358]. Predominant autoantigens, for example, myelin basic protein and myelin oligodendrocyte glycoprotein, are targets for CatS processing in antigen‐presenting cells [358, 359]. Furthermore, altered CatS expression has been linked with disease activity [354]. Finally, a recent study showed that altered expression of cysteine cathepsins mitigates fast endo/lysosomal degradation of the immunodominant epitope 40–48 of myelin oligodendrocyte glycoprotein [360].

### Lysosomal peptidases in brain pathologies related to lysosomal storage disease

Mutations in genes encoding proteins involved in lysosomal function cause lysosomal storage diseases, which are characterized by the progressive accumulation of undegraded substrates inside endo/lysosomal compartments [361, 362]. In the CNS, neuronal ceroid lipofuscinoses (NCLs) are known to be caused by inactivation mutations in Cat genes (Table 2), namely defects in CatD and Cat F (CatF), which result in type 10 and type 13 of NCL, respectively [362]. In particular, NCL10 is caused by mutations in the CatD gene due to autosomal recessive inheritance [363], accompanied by congenital, late infantile, or juvenile onset. To date, 21 mutations have been identified that affect the CatD gene, whereas only nine mutations have been confirmed to be pathogenic and linked to the development of NCL10 (reviewed in [362]). A study on CatF‐deficient mice revealed that CatF is also involved in NCL‐like neurodegenerative disorders, as CatF‐deficient mice developed progressive neurological features with onsets at 12–16 months and died prematurely. Additionally, CatF‐deficient mice accumulated large amounts of autofluorescent lipofuscin in the CNS, which is a characteristic of NCLs [364]. Further studies confirmed that mutations in the CatF gene result in NCL type 13, an adult‐onset form of NCL, also known as type B Kufs disease [365, 366, 367, 368, 369]. To date, nine mutations with recessive inheritance were associated with NCL13, and multiple lines of evidence suggest that CatF variants are indeed pathogenic mutations (reviewed in [362]).

Nevertheless, no human patient with dysfunctional CatB and CatL was identified so far. Like CatD‐deficient mice [370], CatB‐ and CatL‐deficient mice also display pronounced lysosomal storage diseases that lead to extensive neuronal death in the CNS and to the development of pronounced brain atrophy due to massive apoptosis of neurons in the cerebral cortex and cerebellar Purkinje and granule cell layers. However, prior to neuronal cell death, CatB‐ and CatL‐deficient neurons develop a lysosomal storage disease similar to human NCL, suggesting that CatB and CatL are essential for the maturation and integrity of the postnatal CNS [269, 370]. CatB and CatL can compensate for each other *in vivo*, since only CatB^−/−^L^−/−^ double‐mutant mice develop neurodegeneration accompanied by pronounced reactive astrocytosis [269]. Nevertheless, cathepsins have been linked to another progressive lysosomal storage disease, Niemann–Pick disease type C (NPC), characterized by intracellular accumulation and redistribution of cholesterol in a number of tissues, including the brain [371]. The increased levels and activities and altered subcellular distribution of CatB and CatD in the cerebellum of mouse brain with NPC pathology have been associated with the underlying cause of neuronal vulnerability in NPC brains. However, a study by Cermak et al. showed that CatB and CatL, but not CatD, represent major lysosomal peptidases that control lysosomal function. The inhibition of CatB and CatL, but not CatD, leads to lysosomal impairment. Furthermore, loss of CatB and CatL activity leads to the accumulation of free cholesterol in late endo/lysosomes, resembling a phenotype characteristic of Niemann‐Pick disease type C [372].

## Conclusions

Lysosomal peptidases represent a pool of enzymes involved in both intracellular catabolism of waste proteins and important physiological functions, such as apoptosis, processing hormones, activating other enzymes, and maintaining homeostasis of immune and neuronal cells. If lysosomal peptidase activity is not properly controlled, excessive protein degradation may lead to severe cell and tissue damage or changes associated with numerous pathologies, the most investigated being cancer, neurodegeneration, and immune disorders. As tumors progress from transformed cells toward highly malignant cells, they pass through several stages that require the action of peptidases. They induce EMT to the malignant cell phenotype and the escape of cancer cells from the primary site, breaking down connective barriers of the ECM and basement membrane during cell migration and extravasation at distant sites during metastases. Lysosomal peptidases are also involved in mechanisms preventing tumor cell apoptosis and immune surveillance. Conversely, they may promote the antitumor action of cytotoxic immune cells, such as CTLs and NK cells. Lysosomal peptidase dysfunction is also typical for neurodegenerative diseases. It can result in compromised proteolytic degradation of misfolded proteins, formation of amyloid aggregates, neuronal loss, and neuroinflammation. Endogenous protein inhibitors of lysosomal peptidases may counterbalance the harmful proteolytic action during pathological processes; however, they may also affect the processes leading to disease regression, such as antitumor immune responses, tumor cell apoptosis, or dissolving of protein aggregates. The regulation of lysosomal peptidases as a therapeutic approach must be fine‐tuned either by specific peptidase inhibitors or by transcription/translation editing and must focus on the harmful fractions of particular peptidases by using advanced delivery systems.

## Conflicts of interest

There are no conflicts of interest to declare.

## Author contributions

JK and AP designed the concept of the review manuscript. JK, AM, MPN, and AP prepared the draft manuscript. AP and AM prepared Fig. 1. AM prepared Table 1 and designed the graphical abstract. AP prepared Table 2. JK reviewed and edited the manuscript. All authors have read and agreed to the published version of the manuscript.

## Data accessibility

All original data are available from the corresponding author on request.
